# Inhibiting microRNA-200a-3p attenuates pyroptosis via targeting the SIRT1/NF-κB/NLRP3 pathway in H_2_O_2_-induced HAEC

**DOI:** 10.18632/aging.205121

**Published:** 2023-10-23

**Authors:** Jia Liu, Youyou Yan, Dongdong Zheng, Jifeng Zhang, Junnan Wang

**Affiliations:** 1 Department of Cardiology, Second Affiliated Hospital of Jilin University, Changchun 130022, China; 2 Department of Cardiovascular Surgery, Second Affiliated Hospital of Jilin University, Changchun 130022, China; 3 Pharmaceutical Sciences of Jilin University, Changchun 130021, China

**Keywords:** pyroptosis, H_2_O_2_, atherosclerosis, SIRT1, NF-κB

## Abstract

Atherosclerosis is a chronic inflammatory disease of the arterial wall caused by many factors. Endothelial cell dysfunction is the initial factor in the development of atherosclerosis, and ROS activates the assembly of inflammasomes and induces the pyroptosis of vascular endothelial cells. Whether H_2_O_2_ induced human aortic endothelial cells (HAECs) pyroptosis and the underlying mechanisms remain unclear. This study aimed to investigate the role of microRNA-200a-3p in H_2_O_2_-induced HAECs pyroptosis. First, we found that the pyroptosis-related protein was upregulated in aortia in HFD *apoE^-/-^* mice. The *in vitro* study showed that the activation of NLRP3 inflammasomes and the pyroptosis in H_2_O_2_-induced HAECs, which is characterized by an increase in Lactate dehydrogenase (LDH) activity, and an increase in propidium iodide (PI)-positive cells. The expression of silent information regulator of transcription 1 (SIRT1) was also decreased in H_2_O_2_-induced HAECs, and the overexpression of SIRT1 could reverse the occurrence of pyroptosis, partly through p65 deacetylation, thereby inhibiting nuclear translocation of p65 and regulating NLRP3 expression. Further studies revealed increased miRNA-200a-3p expression in H_2_O_2_-induced HAECs and the promotion of pyroptosis, which was achieved by targeting SIRT1. Inhibition of miR-200a-3p reduced pyroptosis by promoting the expression of the downstream target gene SIRT1 and reducing the accumulation of p65 and NLRP3. Collectively, our results suggest that H_2_O_2_ can regulate NLRP3 inflammasomes through the miR-200a-3p/SIRT1/NF-κB (p65) signaling pathway and promote HAEC pyroptosis. The miR-200a-3p inhibitor can promote the expression of SIRT1 and inhibit pyroptosis, which may be important to prevent and treat atherosclerosis.

## INTRODUCTION

Atherosclerosis is a chronic inflammatory disease that can cause cardiovascular disease, stroke, and ischemic gangrene. The pathogenesis of atherosclerosis mainly includes endothelial cell injury response mechanisms, lipid deposition, inflammatory response mechanisms involved in immune cells, and vascular smooth muscle proliferation and migration mechanisms. Vascular endothelial dysfunction caused by endothelial injury not only plays a key role in the early stage of atherosclerosis, but is related to plaque progression and atherosclerotic complications [1]. Various causes of atherosclerosis, including hypercholesterolemia, hyperglycemia, hypertension, and smoking, can cause excessive accumulation of intracellular reactive oxygen species (ROS), acute oxidative stress, endothelial damage, and even endothelial cell death, including apoptosis, pyroptosis, and cell necrosis, eventually leading to endothelial dysfunction and various oxidative stress-related diseases. Therefore, oxidative stress intervention is an effective strategy for preventing and treating endothelial injury in atherosclerosis.

In recent years, pyroptosis has been identified as a form of inflammatory cell death that plays an important role in vascular endothelial dysfunction mediated by oxidative stress. Pyroptosis is one of the important pathways that lead to endothelial cell death, and is closely related to the occurrence and development of atherosclerosis [2]. In the early stages of atherosclerosis, the pyroptosis of endothelial cells leads to loss of cell integrity, resulting in the release of inflammatory factors, increased vascular permeability, and monocyte recruitment. Pyroptosis is characterized by its dependence on caspase-1 and the release of numerous pro-inflammatory factors. Among the classical pathways, the nod-like receptor containing a pyrin domain 3 (NLRP3) inflammasome is the most widely studied. NLRP3 inflammasomes are composed of the intracellular recognition receptor, NLRP3, junction protein (ASC), and an effector protein (caspase-1) [3]. In the typical pyroptosis pathway, an increase in ROS levels in the vascular endothelium promotes the expression of NLRP3, apoptosis-associated speck-like protein containing a CARD (ASC), gasdermin-D (GSDMD), pro-IL-1β, and pro-IL-18. NLRP3 combines with pro-caspase-1 through ASC to form an inflammasome complex, cleaves pro-caspase-1 to form active caspase-1. Notably, activated caspase-1 has a shear function, and can promote the formation of the active peptide, GSDMD, mature IL-1β, and IL-18 with N-terminal domain. In addition, N-GSDMD forms a perforated channel on the cell membrane surface, which promotes the influx of water molecules and the outflow of inflammatory factors to induce an acute inflammatory reaction, finally leading to pyroptosis [4]. H_2_O_2_ is a member of the ROS family, and similar to nitric oxide, can spread rapidly in cells and react with cysteine in the protein and change its function [5]. A small amount of H_2_O_2_ in cells can maintain their physiological state, and excessive accumulation of H_2_O_2_ can cause cell damage by oxidative stress [6], leading to pyroptosis.

Nuclear factor kappa B (NF-κB) is a series of related protein complexes that play transcriptional regulatory roles as heterodimers or homodimers and are widely involved in the occurrence of inflammatory diseases. Numerous studies have shown that NF-κB (p65), as a nuclear transcription factor, may regulate the expression of NLRP3 in the pathogenesis of many inflammatory diseases, such as atherosclerosis [7] and hepatic fibrosis [8]. Therefore, we explored whether NF-κB (p65) is involved in mediating endothelial cell pyroptosis and its regulatory mechanism. NF- κB has been found to participate in and affect a wide range of biological processes. Complex and highly regulated mechanisms exist for the activity control of NF-κB. In particular, acetylation of the p65 subunit plays an important role in regulating its transcriptional activity, while acetylation of lysine 310 is necessary for p65 complete transcriptional activity [9]. Previous studies revealed that silent information regulator of transcription 1 (SIRT1) inhibits the transcription of NF-κB by directly deacetylating the p65 protein at lysine 310 [10]. SIRT1 is an NAD^+^-dependent histone deacetylase and a key regulator of oxidative stress [11]. SIRT1 can remove acetyl groups from many histone and non-histone proteins, including the important transcription factors, FOXO3, p53, and NF-κB [12, 13]. SIRT1 regulates key metabolic processes, including gene silencing, stress resistance, apoptosis, senescence, and inflammation, through the deacetylation of different substrates [14, 15]. However, whether SIRT1 is involved in H_2_O_2_-induced inflammation and pyroptosis of human aortic endothelial cells (HAECs) remains unknown. We aimed to determine whether SIRT1 affects the transcriptional activity of NF-κB by regulating the acetylation level of NF-κB (p65) and inhibiting the nuclear translocation of p65 during oxidative stress in endothelial cells. Such findings may help us better understand the pathogenesis of pyroptosis in atherosclerosis.

MicroRNAs (miRNAs) are endogenous non-coding RNAs (ncRNAs) approximately 18–24 nucleotides in length. miRNAs have attracted extensive attention as potential therapeutic targets for atherosclerosis-related diseases [16, 17]. The main role of miRNAs is to negatively regulate gene expression by binding to the target mRNA, inducing its degradation or inhibiting its translation [18]. When miRNAs cannot achieve complete complementarity or when they bind to the 5'UTR of the target gene, they inhibit translation and play an important role in regulating protein synthesis [19, 20]. miRNAs act as signaling molecules to transmit genetic information between cells and tissues [21]. Some physiological processes and pathological results are highly dependent on miRNAs, including cancer, cardiovascular diseases, and nervous system diseases [22]. The mechanisms related to miRNAs are helpful for the treatment of related diseases. MiR-200a-3p is widely expressed in various tissues. Further, studies have shown that miR-200a-3p has a regulatory effect on different cardiovascular diseases. To explore the upstream regulatory mechanism affecting the expression of SIRT1 and the technical means to interfere with SIRT1 regulation of pyroptosis, bioanalysis predicted that a target may exist between miR-200a-3p and SIRT1, and miR-200a-3p may affect the occurrence of pyroptosis by regulating the expression of SIRT1. In this study, human aortic endothelial cells (HAECs) were used as the research object to examine the regulation and mechanism of NF-κB (p65), SIRT1, and miR-200a-3p in the pyroptosis of the HAECs oxidative stress model. The findings of this study provide a new theoretical basis for the role of p65, SIRT1, and miR-200a-3p in oxidative stress and may provide a new strategy for the clinical prevention and treatment of atherosclerosis.

## RESULTS

### Oxidative stress induces HAEC injury

**Figure 1:**
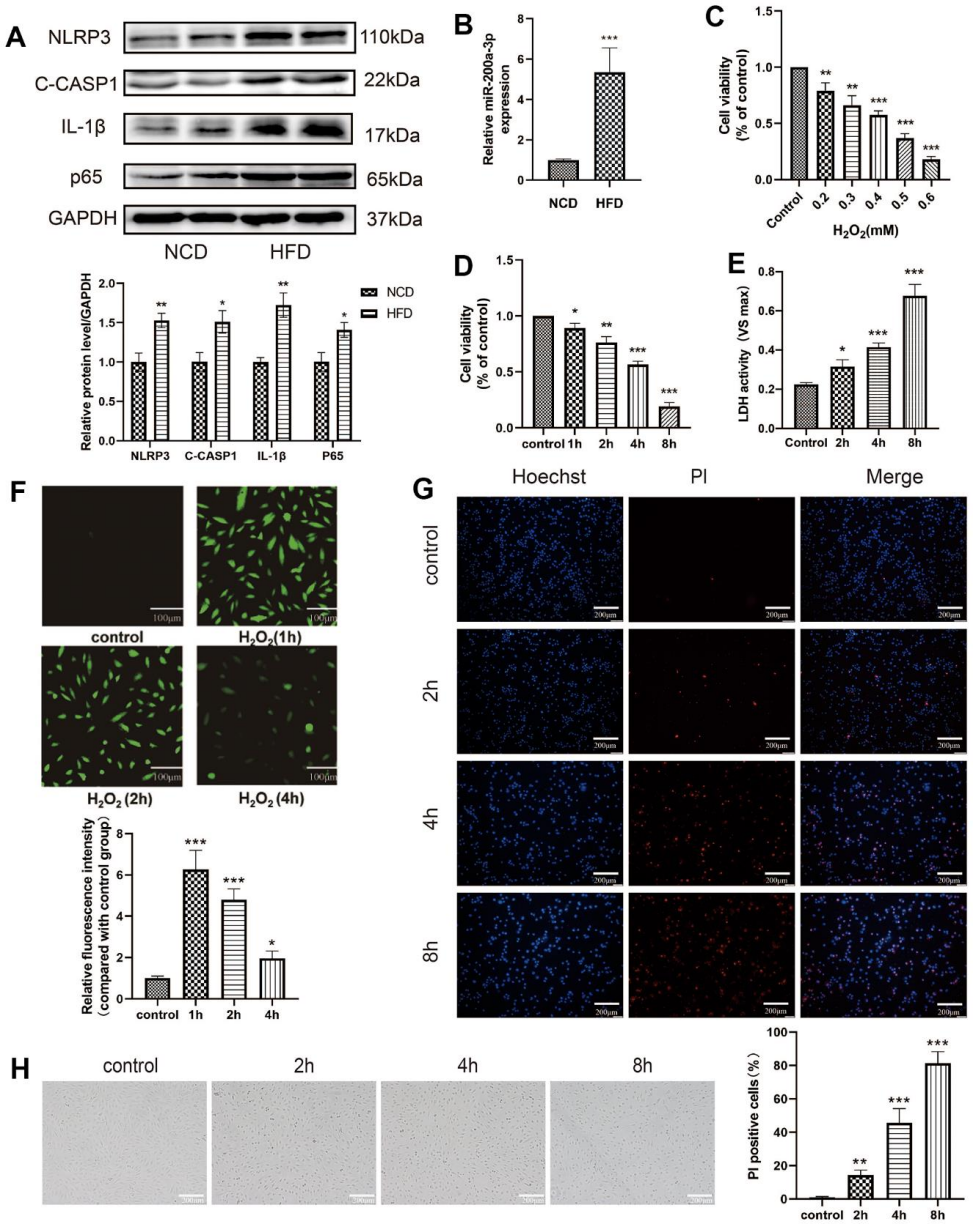
**Oxidative stress induces HAEC injury.** (**A**) The pyroptosis-related proteins were assessed via western blot in aortia in *apoE^-/-^* mice. (**B**) qRT-PCR results showing the miRNA-200a-3p expression increased in HFD group compared with that in NCD group. (**C**) HAECs were treated with different concentrations of H_2_O_2_ (0.2 mM, 0.3 mM, 0.4 mM, 0.5 mM, 0.6 mM) for 4 h, and cell viability was detected using the CCK-8 assay. (**D**) HAECs were treated with 0.4 mM H_2_O_2_ for different durations (0 h, 1 h, 2 h, 4 h, 8 h), and cell viability was detected using the CCK-8 assay. (**E**) LDH released from HAECs treated with H_2_O_2_ (0.4 mM) at different times. (**F**) H_2_O_2_ stimulation significantly increased intracellular ROS, which contributed to the induction of cell damage. Plotting scale =100 μm. (**G**) PI staining results revealed increased percentage of PI positive cells in H_2_O_2_-treated HAECs in a time-dependent manner. Double staining of PI (red) and Hoechst 33342 (blue). Plotting scale = 200 μm. (**H**) Morphological changes of HAECs under light microscope. Plotting scale = 200 μm. (*p<0.05 vs control, **p<0.01 vs control, ***p<0.001 vs control). Data are presented as mean ± SD (n=3). HAECs, human aortic endothelial cells; CCK-8, cell-counting kit 8; LDH, lactate dehydrogenase; ROS, reactive oxygen species; PI, propidium iodide; SD, standard deviation.

In this study, we first found that the expression of the pyroptosis-related protein was upregulated in aortia in HFD group (Figure 1A), and the miRNA-200a-3p level was significantly increased in HFD group (Figure 1B). In order to explore the mechanism, *in vitro* oxidative stress response was stimulated by cell treatment with H_2_O_2._ HAECs were treated with different concentrations of H_2_O_2_ (0.2 mM, 0.3 mM, 0.4 mM, 0.5 mM, 0.6 mM) for 4 h, and cell viability was determined using the CCK-8 assay (Figure 1C). HAECs were treated with 0.4 mM H_2_O_2_ for different durations (0 h, 1 h, 2 h, 4 h, 8 h), and cell viability was detected using the CCK-8 assay (Figure 1D). LDH was released from HAECs treated with H_2_O_2_ (0.4 mM) at different times (Figure 1E). H_2_O_2_ stimulation also significantly increased intracellular ROS levels, contributing to the induction of cell damage (Figure 1F). Further, PI staining results revealed that the percentage of PI-positive cells increased in H_2_O_2_-treated HAECs in a time-dependent manner (Figure 1G). The changes of HAECs swelling and transparency of its cytoplasm can be seen with light microscope (Figure 1H).

### Oxidative stress induces pyroptosis of HAECs

**Figure 2:**
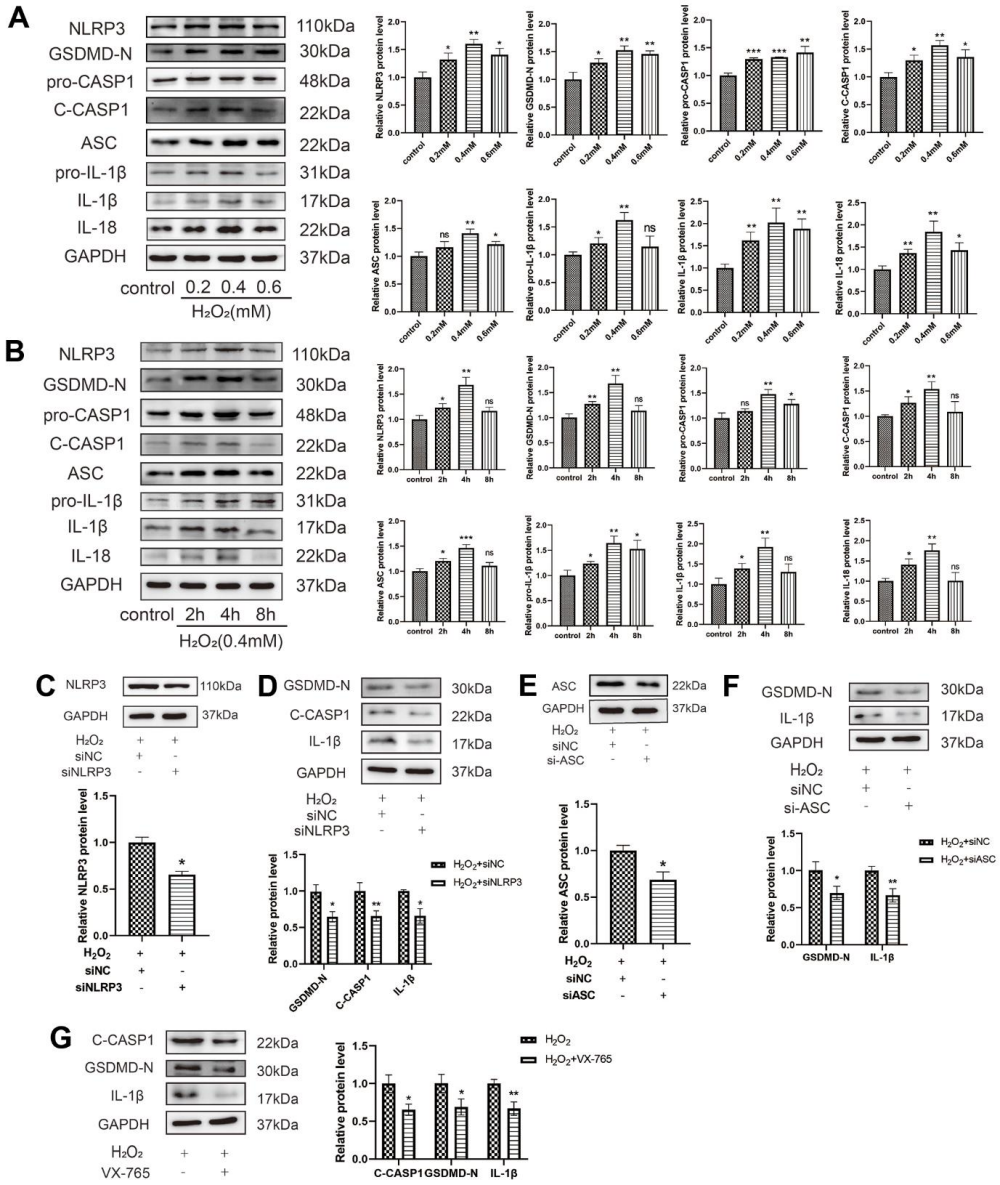
**Oxidative stress induces HAEC pyroptosis.** (**A**) HAECs were treated with 0, 0.2, 0.4, or 0.6 mM H_2_O_2_ for 4 h, and the pyroptosis-related proteins were assessed via western blot analysis. H_2_O_2_-induced HAEC pyroptosis in a dose-dependent manner. (**B**) HAECs were treated with H_2_O_2_ (0.4 mM) for different durations (0 h, 2 h, 4 h, 8 h) and the pyroptosis-related proteins were assessed via western blot. H_2_O_2_ induced HAEC pyroptosis in a time-dependent manner. (**C**) Western blot revealing the downregulation of the NLRP3 protein in HAECs treated with siNLRP3 via transient transfection. (**D**) Western blot demonstrating the decreased protein levels of GSDMD-N, C-caspase-1, and IL-1β in HAECs transduced with siNLRP3 compared with cells transduced with NC under H_2_O_2_ treatment. (**E**) Western blot demonstrating the downregulation of the ASC protein in HAECs treated with siASC via transient transfection. (**F**) Western blot demonstrating the decreased protein levels of GSDMD-N and IL-1β in HAECs transduced with si-ASC compared with cells transduced with NC under H_2_O_2_ treatment. (**G**) Western blot demonstrating the decreased protein levels of C-caspase-1, GSDMD-N, and IL-1β in HAECs treated with VX-765 compared with cells without VX-765 under H_2_O_2_ treatment. Data are presented as mean ± SD (n=3). (^*^p<0.05 vs control, H_2_O_2_ or H_2_O_2_+siNC, ^**^p<0.01 vs control, H_2_O_2_ or H_2_O_2_+siNC, ^***^p<0.001 vs control, ns, no significance). HAECs, human aortic endothelial cells; NC, negative control.

Pyroptosis is a novel form of cell death that is mediated by the inflammasome and caspase-1 activation. To determine whether H_2_O_2_ could induce the pyroptosis of HAECs, HAECs were treated with different concentrations of H_2_O_2_ for 4 h. Western blot analysis revealed that H_2_O_2_ upregulated the expression of the pyroptosis-related proteins in HAECs in a dose-dependent manner (Figure 2A). When HAECs were treated with H_2_O_2_ (0.4 mM) at different times, western blot analysis also revealed that H_2_O_2_ upregulated the expression of the pyroptosis-related proteins in HAECs (Figure 2B). To further determine whether H_2_O_2_-induced pyroptosis of HAECs is dependent on NLRP3 inflammation, a transfection experiment was conducted. siNLRP3 was found to decrease the expression levels of GSDMD-N, C-caspase-1, and IL-1β in HAECs treated with H_2_O_2_ (Figure 2C, 2D). Further, si-ASC decreased the expression levels of GSDMD-N and IL-1β in HAECs treated with H_2_O_2_ (Figure 2E, 2F). To investigate whether H_2_O_2_-induced pyroptosis of HAECs is caspase-1 dependent, a caspase-1 inhibitory experiment was conducted. The caspase-1 selective inhibitor, VX-765, decreased the level of activated caspase-1 and inhibited the maturation of IL-18 and IL-1β (Figure 2G). Thus, H_2_O_2_-induced pyroptosis of HAECs is dependent on NLRP3 inflammation.

### Downregulation of p65 abolishes H_2_O_2_-induced pyroptosis of HAECs

**Figure 3:**
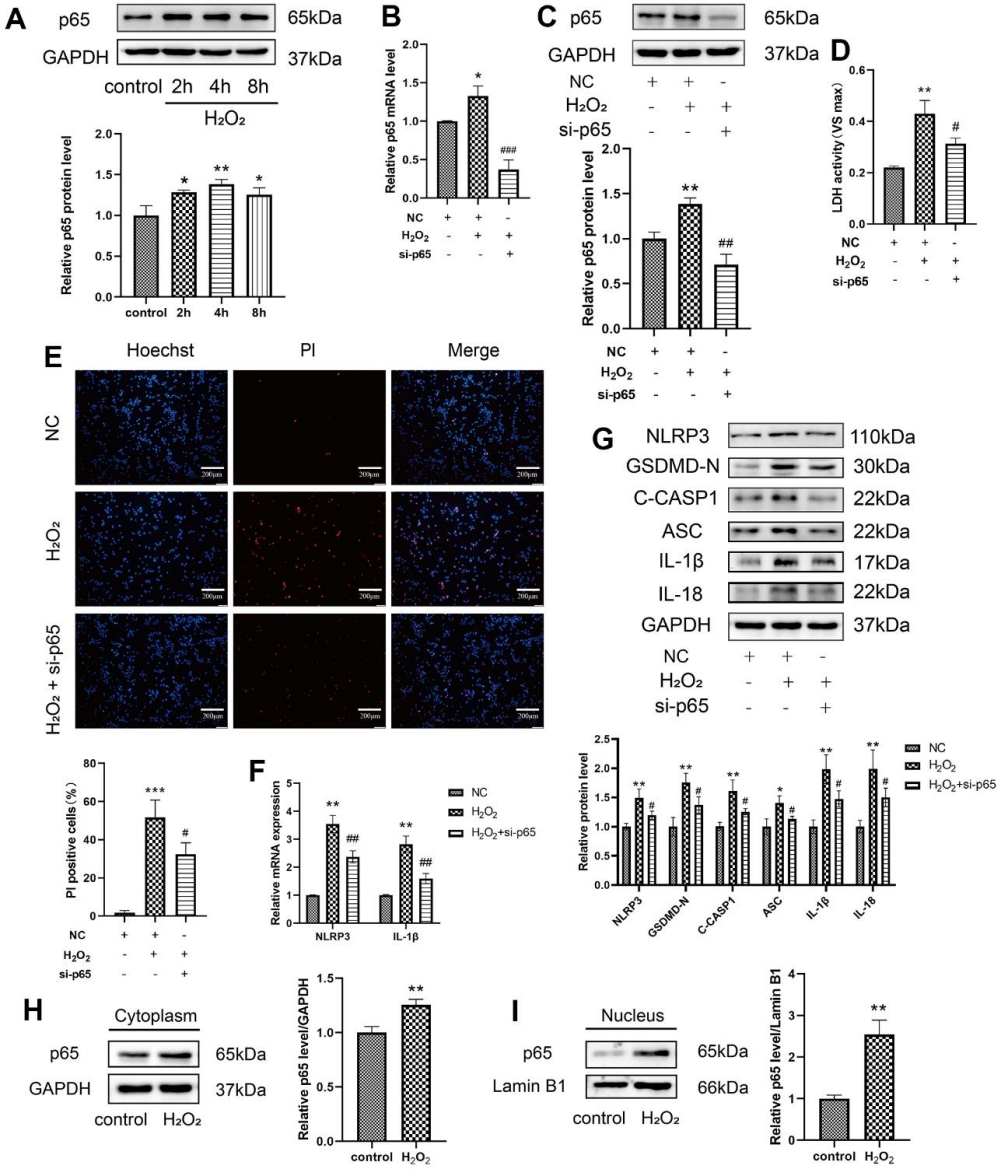
**Effect of p65 on the H_2_O_2_-induced pyroptosis of HAECs.** (**A**) Western blot demonstrating the increased protein levels of p65 upon H_2_O_2_ treatment in a time-dependent manner. (**B**) qRT-PCR results demonstrating the decreased mRNA expression of p65 in cells transduced with si-p65 compared with that in untransduced cells. (**C**) Western blot demonstrating the decreased protein expression of p65 in cells transduced with si-p65 compared with that in untransduced cells. (**D**) LDH release from HAECs treated with or without si-p65 following H_2_O_2_ treatment. (**E**) PI staining results of the increased percentage of PI positive cells in H_2_O_2_-treated HAECs. Double staining of PI (red) and Hoechst 33342 (blue). The percentage of PI positive cells in H_2_O_2_-treated HAECs decreased after transfection with si-p65. Plotting scale = 200 μm. (**F**) qRT-PCR results demonstrating the increased mRNA expression of NLRP3 and IL-1β in HAECs under H_2_O_2_ treatment, and decreased mRNA expression of NLRP3 and IL-1β in HAECs transduced with si-p65 compared with cells transduced with NC under H_2_O_2_ treatment. (**G**) Western blot results demonstrating the decreased protein expression of NLRP3, GSDMD-N, C-caspase-1, ASC, IL-1β, and IL-18 in HAECs transduced with si-p65 compared with cells transduced with NC under H_2_O_2_ treatment. Western blot demonstrating the increased protein expression of p65 in the cytoplasm (**H**) and nucleus (**I**) of H_2_O_2_-treated HAECs compared with NC-treated cells. The p65 protein levels in nucleus were obviously increased. (^*^p<0.05 vs control or NC, ^**^p<0.01 vs control or NC, ^***^p<0.001 vs NC, ^#^*p*<0.05 vs H_2_O_2_, *^##^p*<0.01 vs H_2_O_2_*. ^###^p*<0.001 vs H_2_O_2_). Data are presented as mean ± SD (n=3). HAECs, human aortic endothelial cells; LDH, lactate dehydrogenase; qRT-PCR, quantitative real-time polymerase chain reaction; PI, propidium iodide; NC, negative control.

p65 is known as an important transcription factor that binds to the promoter regions of target genes and mediates their transcription. Previous studies revealed that NF-κB activation is necessary for NLRP3 activation [23]. To explore whether p65 directly affects NLRP3 expression in H_2_O_2_-induced pyroptosis, the protein levels of p65 were first found to increase after H_2_O_2_ treatment in a time-dependent manner (Figure 3A). The effect of p65 deficiency on H_2_O_2_-induced pyroptosis was assessed. siRNA targeting p65 was used to reduce the protein expression of p65. Remarkably, si-p65 decreased H_2_O_2_-induced upregulation of LDH release from HAECs and the percentage of PI-positive cells (Figure 3D, 3E). To explore the role of p65 and NLRP3 in H_2_O_2_-induced pyroptosis, p65 was silenced with siRNA. The mRNA expression levels of NLRP3 and IL-1β decreased in HAECs transduced with si-p65 compared to those treated with H_2_O_2_ (Figure 3F). In addition, si-p65 decreased the H_2_O_2_-induced upregulation of NLRP3, GSDMD-N, C-caspase-1, ASC, IL-1β, and IL-18 protein in HAECs (Figure 3G). Nuclear plasma separation was performed to assess protein distribution in the cell nucleus and cytoplasm. Based on western blotting, H_2_O_2_ promoted the expression of p65 in the cytoplasm and nucleus (Figure 3H, 3I). In fact, the p65 protein levels in the nucleus were obviously increased.

### SIRT1 overexpression abolishes H_2_O_2_-induced pyroptosis of HAECs

**Figure 4:**
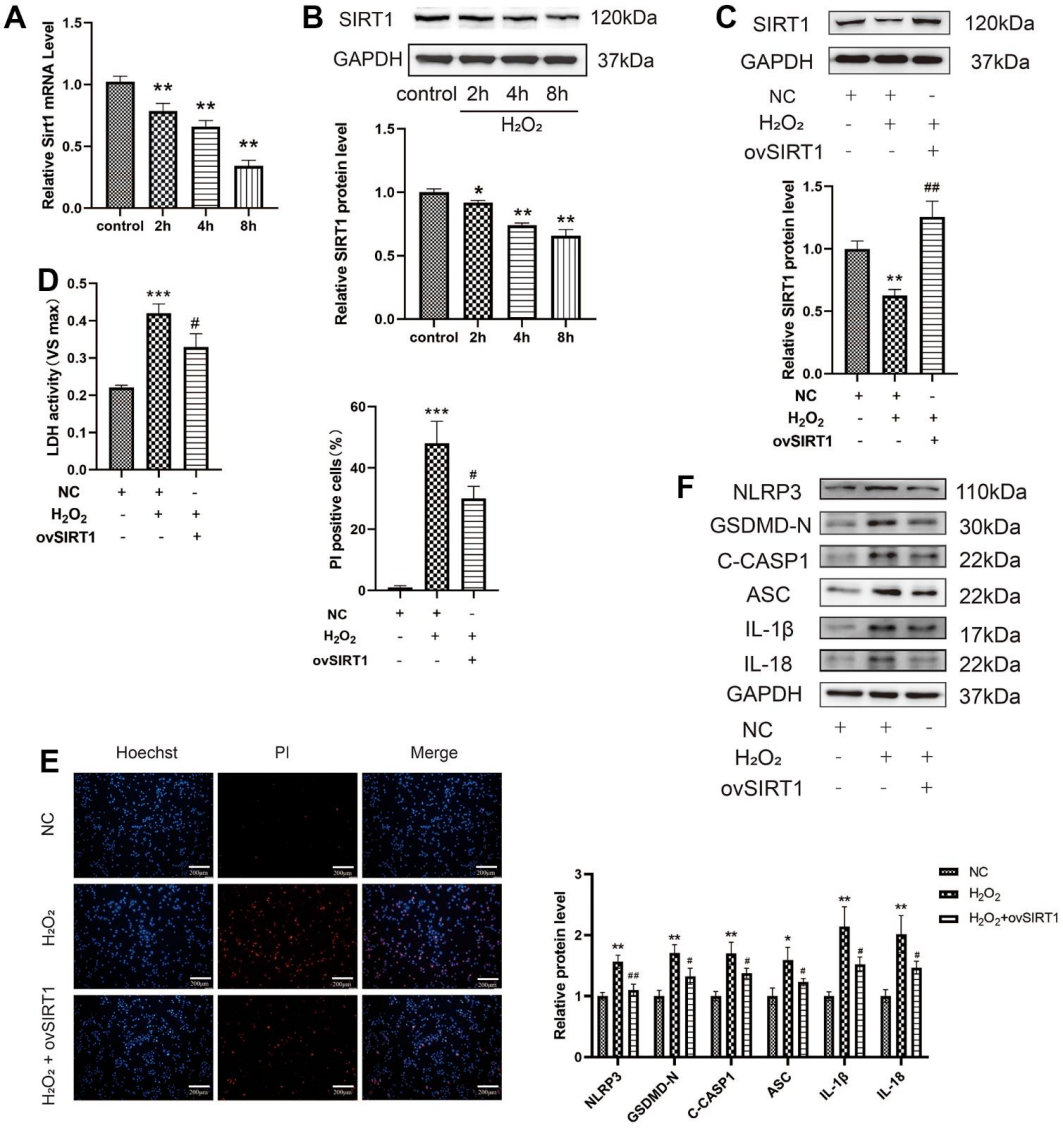
**Effect of SIRT1 on the H_2_O_2_-induced pyroptosis of HAECs.** (**A**) qRT-PCR results demonstrating the decreased mRNA expression of Sirt1 upon H_2_O_2_ treatment in a time-dependent manner. (**B**) Western blot demonstrating the decreased protein levels of SIRT1 upon H_2_O_2_ treatment in a time-dependent manner. (**C**) Western blot demonstrating the overexpression of the SIRT1 protein in HAECs treated with ov-SIRT1 via transient transfection. (**D**) LDH release from HAECs treated with or without ov-SIRT1 following H_2_O_2_ treatment. (**E**) PI staining results demonstrating the increased percentage of PI positive cells in H_2_O_2_-treated HAECs. Double staining of PI (red) and Hoechst 33342 (blue). The percentage of PI positive cells in H_2_O_2_-treated HAECs decreased after transfection with ov-SIRT1. Plotting scale = 200 μm. (**F**) Western blot demonstrating the increased ratio of NLRP3, GSDMD-N, C-caspase-1, ASC, IL-1β, IL-18 in cells with H_2_O_2_ treatment compared with cells without H_2_O_2_ treatment. The ratio of NLRP3, GSDMD-N, C-caspase-1, ASC, IL-1β, and IL-18 decreased in cells transduced with ov-SIRT1 under H_2_O_2_ treatment compared with cells transduced with NC. (^*^p<0.05 vs control or NC, ^**^p<0.01 vs control or NC, ^***^p<0.001 vs NC, ^#^*p*<0.05 vs H_2_O_2_, *^##^p*<0.01 vs H_2_O_2_). Data are presented as mean ± SD (n=3). HAECs, human aortic endothelial cells; qRT-PCR, quantitative real-time polymerase chain reaction; LDH, lactate dehydrogenase; PI, propidium iodide; GSDMD, gasdermin-D; ASC, apoptosis-associated speck-like protein containing a CARD; NC, negative control.

Further exploration of the molecular mechanisms of p65-mediated pyroptosis revealed that acetylation of the p65 subunit plays an important role in the regulation of NF-κB transcriptional activity. SIRT1 inhibits the transcription of NF-κB by deacetylating the p65 protein at lysine 310; however, its role in HAEC pyroptosis has not been studied. The mRNA and protein levels of Sirt1 decreased after H_2_O_2_ treatment in a time-dependent manner (Figure 4A, 4B). The overexpression of SIRT1 in HAECs via transduction with the SIRT1 plasmid is shown in Figure 4C. SIRT1 overexpression decreased LDH release from HAECs treated with H_2_O_2_ and the percentage of PI-positive cells (Figure 4D, 4E). Moreover, SIRT1 overexpression decreased the protein expression of NLRP3, GSDMD-N, C-caspase-1, ASC, IL-1β, and IL-18 in HAECs treated with H_2_O_2_ (Figure 4F). Overall, SIRT1 protects HAECs from H_2_O_2_-induced injury.

### SIRT1 overexpression abolishes pyroptosis by deacetylating p65 in H_2_O_2_-induced pyroptosis of HAECs

**Figure 5:**
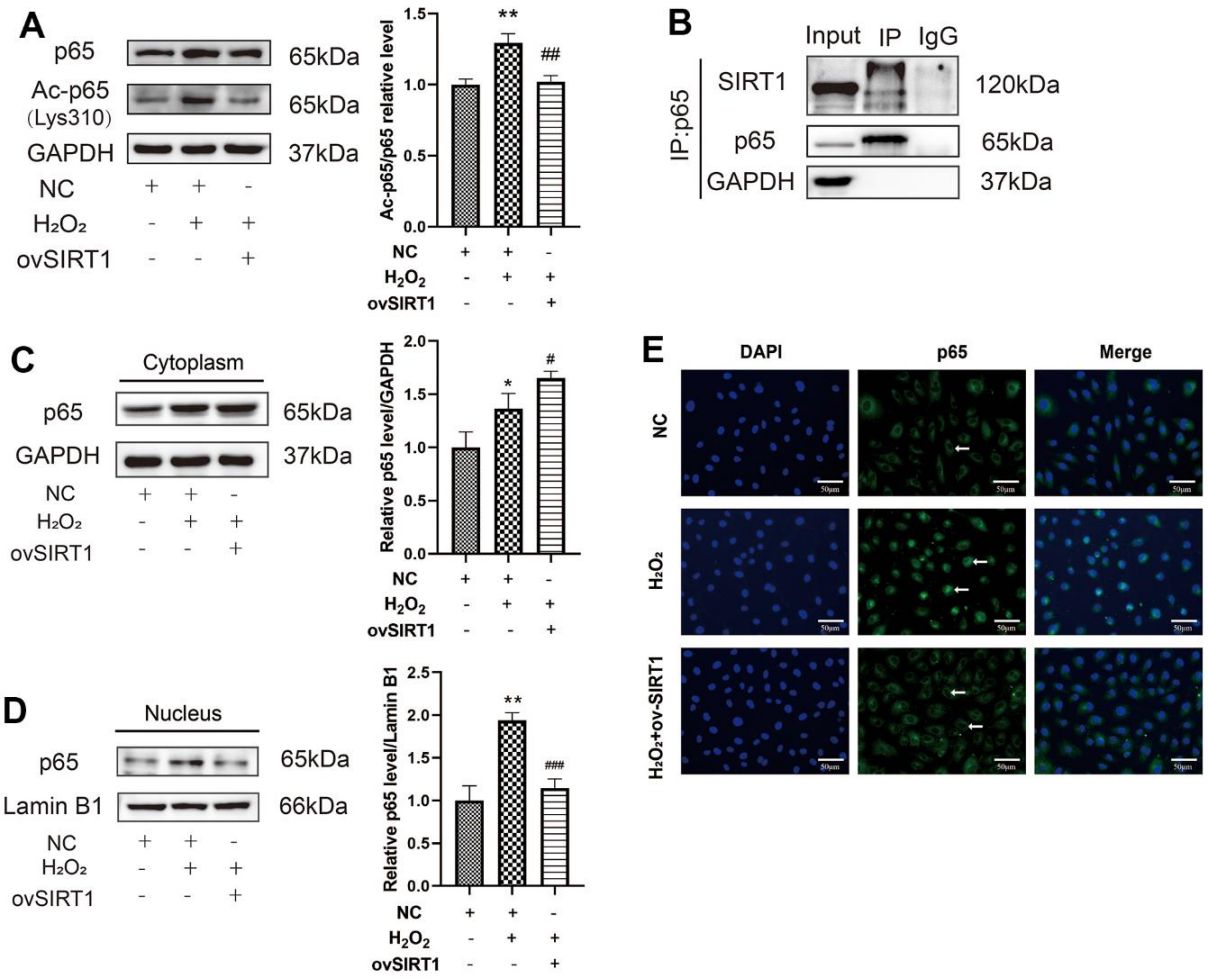
**Effect of SIRT1 deacetylation on p65 in H_2_O_2_-induced pyroptosis of HAECs.** (**A**) Western blot demonstrating the increased protein expression ratio of Ac-p65 (Lys310)/p65 in the H_2_O_2_ group compared with the NC group. The overexpression of SIRT1 protein levels decreased the protein expression ratio of Ac-p65 (Lys310)/p65 compared with that in cells treated with H_2_O_2_. (**B**) Endogenous IP Western revealed the pulling down of the HAEC extracts with either a negative control (IgG) or RelA/p65 antibody, followed by SIRT1 or RelA/p65 immunoblot. (**C**) Western blot demonstrating the increased p65 protein levels in the cytoplasm of H_2_O_2_-treated HAECs compared with NC-treated cells. Overexpression of SIRT1 protein levels increased the p65 protein levels in the cytoplasm compared with that in cells treated with H_2_O_2_. (**D**) Western blot demonstrating increased p65 protein levels in the nucleus of H_2_O_2_-treated HAECs compared with NC-treated cells. Overexpression of SIRT1 protein levels decreased p65 protein levels in the nucleus compared with that in cells treated with H_2_O_2_. (**E**) Double staining of p65 (green) and DAPI (blue). DAPI staining results of the nucleus of HAECs. P65 staining results of increased p65 in the nucleus of H_2_O_2_-treated HAECs; the level decreased after transfection with ov-SIRT1 in H_2_O_2_-treated HAECs under a fluorescence microscope. Plotting scale =50 μm. (^*^p<0.05 vs NC, ^**^p<0.01 vs NC, ^#^*p*<0.05 vs H_2_O_2_, *^##^p*<0.01 vs H_2_O_2_). Data are presented as mean ± SD (n=3). HAECs, human aortic endothelial cells; NC, negative control; IP, immunoprecipitation; DAPI, 4′,6-diamidino-2-phenylindole.

To further verify the relationship between SIRT1 and p65 in H_2_O_2_-induced HAEC pyroptosis, p65 acetylation levels in H_2_O_2_-induced HAECs were first determined. The protein ratio of Ac-p65 (Lys310)/p65 increased in H_2_O_2_-induced HAECs. Further, the overexpression of SIRT1 protein levels decreased the protein expression ratio of Ac-p65 (Lys310)/p65 in H_2_O_2_-induced HAECs (Figure 5A). To determine whether SIRT1 and p65 interact directly, endogenous IP western blotting was performed. Based on the result, the HAECs extracts were pulled down with the p65 antibody and the protein expression of SIRT1 was high (Figure 5B). To clarify whether SIRT1 affects the nuclear translocation of p65, western blotting was performed. SIRT1 overexpression increased p65 protein levels in the cytoplasm and decreased p65 protein levels in the nucleus (Figure 5C, 5D). The p65 staining results also showed that the level of p65 in the nucleus increased in H_2_O_2_-treated HAECs. However, ov-SIRT1 decreased the level of p65 in the nucleus in H_2_O_2_-treated HAECs (Figure 5E). Based on these results, SIRT1 overexpression abolished the inhibitory effect of H_2_O_2_ on HAEC pyroptosis by deacetylating p65 and inhibiting nuclear translocation.

### Downregulation of miRNA-200a-3p abolishes the effect of H_2_O_2_ on pyroptosis

**Figure 6:**
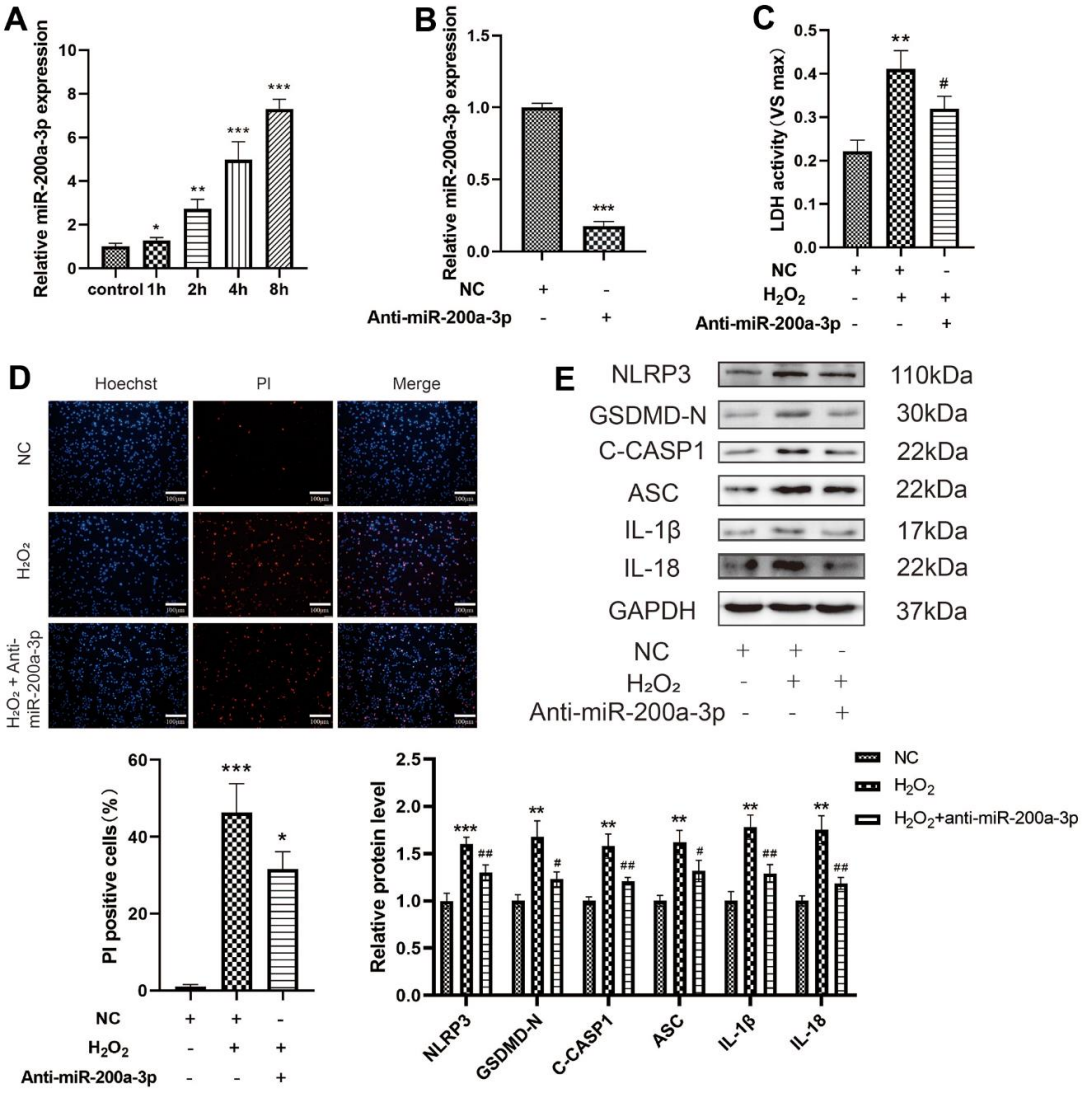
**Effect of miRNA-200a-3p on H_2_O_2_-induced pyroptosis of HAECs.** (**A**) HAECs were treated with H_2_O_2_ (0.4 mM) for 1 h, 2 h, 4 h, and 8 h; miRNA-200a-3p expression increased in all cases compared with that in control cells. (**B**) qRT-PCR results showing the decreased expression of miR-200a-3p in HAECs transduced with the miR-200a-3p inhibitor compared with negative control cells. (**C**) LDH release from HAECs treated with H_2_O_2_ and transduced with or without the miR-200a-3p inhibitor. (**D**) PI staining results showing the decreased percentage of PI positive cells in HAECs transduced with the miR-200a-3p inhibitor. (**E**) Western blot demonstrating increased ratio of NLRP3, GSDMD-N, C-caspase-1, ASC, IL-1β, IL-18 in cells treated with H_2_O_2_ compared with cells that were not treated with H_2_O_2_. The ratio of NLRP3, GSDMD-N, C-caspase-1, ASC, IL-1β, IL-18 decreased in cells transduced with the miR-200a-3p inhibitor under H_2_O_2_ treatment compared with cells transduced with the NC inhibitor. (^*^p<0.05 vs control, ^**^p<0.01 vs control or NC, ^***^p<0.001 vs control or NC*, ^#^p*<0.05 vs H_2_O_2_*, ^##^p*<0.01 vs H_2_O_2_)_._ Data are presented as mean ± SD (n=3). HAECs, human aortic endothelial cells; qRT-PCR, quantitative real-time polymerase chain reaction; LDH, lactate dehydrogenase; PI, propidium iodide; NC, negative control.

To identify an effective target for anti-atherosclerosis therapy through SIRT1, we analyzed the possible direct targets of miR-200a-3p and SIRT1 using bioinformatics. First, the miRNA-200a-3p levels were found to be significantly increased in H_2_O_2_-induced HAECs in a time-dependent manner (Figure 3A). When the effect of miRNA-200a-3p on H_2_O_2_-induced pyroptosis was examined, the miR-200a-3p inhibitor was found to decrease the level of LDH release from HAECs treated with H_2_O_2_ and the percentage of PI-positive cells (Figure 6C, 6D). The miR-200a-3p inhibitor also decreased the protein expression of NLRP3, GSDMD-N, C-caspase-1, ASC, IL-1β, and IL-18 in H_2_O_2_-induced HAECs (Figure 6E)_._ These results indicate that the miR-200a-3p inhibitor protected HAECs from H_2_O_2_-induced injury.

### Downregulation of miRNA-200a-3p abolishes the effect of H_2_O_2_ on the pyroptosis of HAECs via target regulating SIRT1

**Figure 7:**
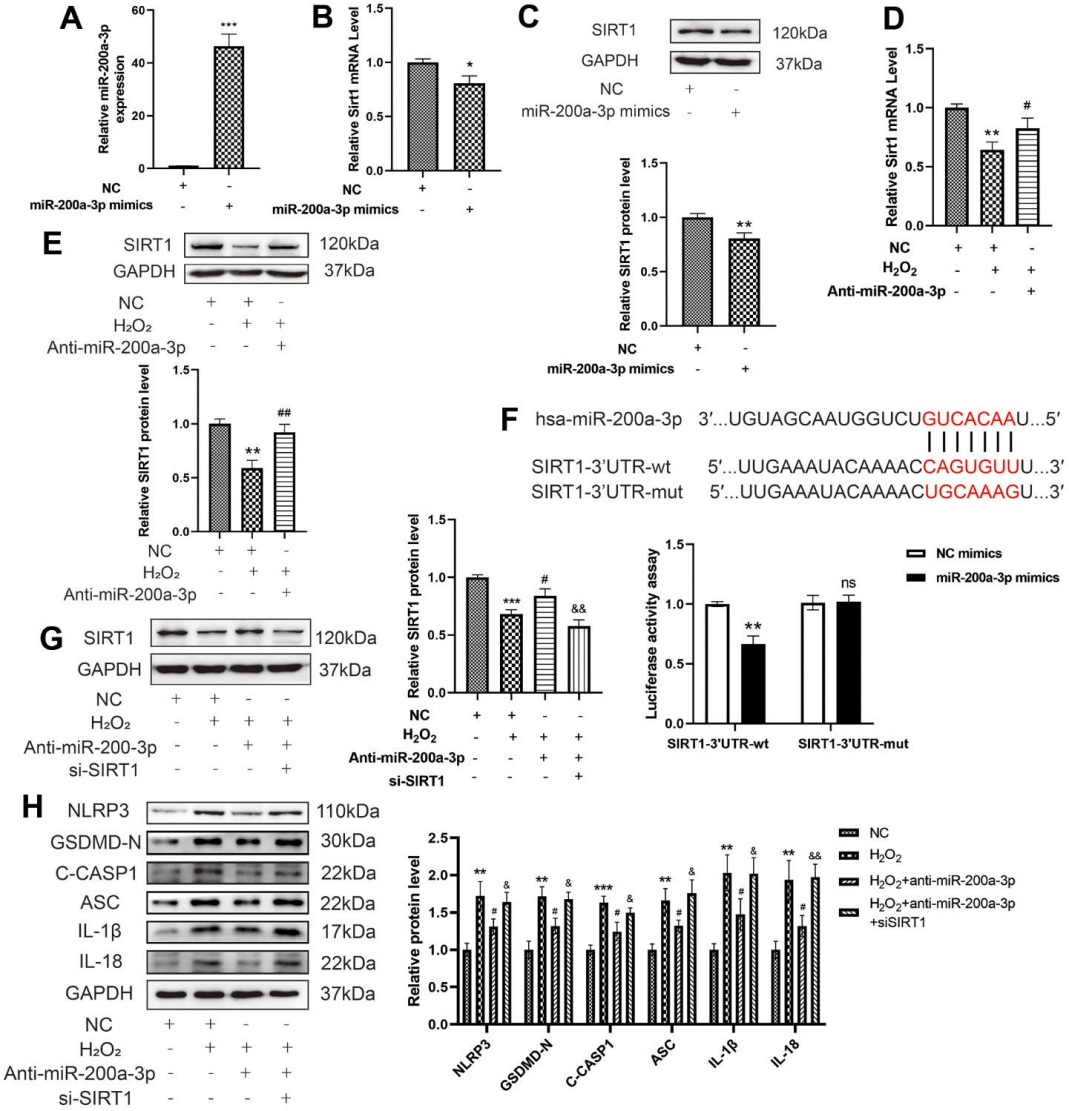
**Effect of miRNA-200a-3p on H_2_O_2_-induced pyroptosis of HAECs via SIRT1.** (**A**) qRT-PCR results showing the increased expression of miR-200a-3p in HAECs transduced with the miR-200a-3p mimics compared with negative control cells. (**B**, **C**) qRT-PCR and western blot results demonstrating the decreased SIRT1 RNA and protein expression in HAECs transduced with miR-200a-3p mimics. (**D**, **E**) qRT-PCR results showing the decreased SIRT1 RNA and protein expression in HAECs treated with H_2_O_2_ compared with cells that were not treated with H_2_O_2_. SIRT1 expression increased in HAECs transduced with the miR-200a-3p inhibitors compared with cells transduced with the NC inhibitor under H_2_O_2_ treatment. (**F**) Schematic of the complementary miR-200a-3p and SIRT1-3'UTR sequences. HEK-293T cells were co-transfected with wild-type or mutant SIRT1-3'UTR reporter constructs and miR-200a-3p mimics or corresponding negative controls (^**^p<0.01 vs SIRT1-3'UTR-wt+NC mimics group*,* ns, not significant vs SIRT1-3'UTR-mut+NC mimics group)_._ (**G**) Western blot demonstrating the decreased expression of SIRT1 in HAECs compared with that in negative control cells with or without H_2_O_2_ treatment. (**H**) Western blot demonstrating the decreased ratio of NLRP3, GSDMD-N, C-caspase-1, ASC, IL-1β, and IL-18 in cells transduced with miR-200a-3p inhibitor under H_2_O_2_ treatment compared with that in cells transduced with the NC inhibitor. The ratio of NLRP3, GSDMD-N, C-caspase-1, ASC, IL-1β, and IL-18 increased in cells transduced with the miR-200a-3p inhibitor and si-SIRT1 under H_2_O_2_ treatment compared with that in cells transduced with the miR-200a-3p inhibitor. (^*^p<0.05 vs NC, ^**^p<0.01 vs NC, ^***^p<0.001 vs NC, *^#^p*<0.05 vs H_2_O_2,_ *^##^p*<0.01 vs H_2_O_2_, *^&^p*<0.05 vs H_2_O_2_+Anti-miR-200a-3p, *^&&^p*<0.01 vs H_2_O_2_+ Anti-miR-200a-3p.). Data are presented as mean ± SD (n=3). HAECs, human aortic endothelial cells; qRT-PCR, quantitative real-time polymerase chain reaction; NC, negative control.

To explore the relationship between miR-200a-3p and SIRT1 in H_2_O_2_-induced HAECs, miR-200a-3p was overexpressed in HAECs via transduction with miR-200a-3p mimics as depicted in Figure 7A. The miR-200a-3p mimics decreased the RNA and protein expression of SIRT1 in HAECs (Figure 7B, 7C), whereas the miR-200a-3p inhibitors increased the RNA and protein expression of SIRT1 in HAECs treated with H_2_O_2_ (Figure 7D, 7E). Based on bioinformatics and the existing literature, the relationship between miR-200a-3p and SIRT1 in H_2_O_2_-induced HAEC pyroptosis was assessed. A schematic representation of complementary miR-200a-3p and SIRT1-3'UTR sequences is provided in Figure 7F. The letters in red indicate the matched bases. By performing a luciferase reporter gene assay, we found that the miR-200a-3p mimic inhibited the expression of SIRT1. Finally, a rescue experiment was performed to verify the direct targeting of SIRT1 by miR-200a-3p. Western blot revealed the decreased expression of SIRT1 in HAECs (Figure 7G). Further, knockdown of miR-200a-3p was found to decrease the expression of NLRP3, GSDMD-N, C-caspase-1, ASC, IL-1β, and IL-18 in HAECs treated with H_2_O_2_. Knockdown of SIRT1 reversed the function of the miR-200a-3p inhibitors, which increased the expression of NLRP3, GSDMD-N, C-caspase-1, ASC, IL-1β, and IL-18 in HAECs treated with H_2_O_2_ (Figure 7H). Overall, miR-200a-3p can regulate SIRT1 expression at the post-transcriptional level by targeting the SIRT1 -3'UTR.

## DISCUSSION

Endothelial cell dysfunction is the initial factor in the development of atherosclerosis, and oxidative stress is an important cause of endothelial injury. According to previous studies, excessive accumulation of ROS can activate inflammasomes, cause endothelial cell pyroptosis, induce endothelial dysfunction [24], and promote the formation of atherosclerosis. In our study, the expression of pyroptosis related protein increased in the aorta in mice with high fat diet. To explore the mechanism, we did the further study *in vitro*. We found that after HAECs were induced by H_2_O_2_ (0.4 mM for 4 h), the cell damage was close to half of the lethal dose, and the expression of the pyroptotic proteins, NLRP3, ASC, pro-caspase-1, C-caspase-1, GSDMD-N, IL-1β, and IL-18 increased significantly. ROS expression was the highest at 1 h after H_2_O_2_ induction, indicating that the cells responded rapidly to H_2_O_2_ stimulation. ROS was also recognized to be involved in mediating HAECs pyroptosis. Through the downregulation of NLRP3 and ASC, and the use of caspase-1 inhibitor VX-765, the occurrence of HAECs pyroptosis decreased, which proved that H_2_O_2_ promoted the occurrence of HAEC pyroptosis and aggravated endothelial cell injury by activating the formation of the NLRP3 inflammasome complex.

To explore the molecular mechanism for the pyroptosis of HAECs, further experiments were performed. Prior studies revealed that NF-κB, a heterodimer and homodimer, plays a transcriptional regulatory role and is involved in the occurrence of inflammatory diseases. We explored whether p65 plays a role in regulating the expression level of NLRP3 in HAECs. After low expression of p65, the activity of LDH and the positive rate of PI staining in HAECs induced by H_2_O_2_ decreased. Further, the expression levels of the pyroptosis-related proteins, NLRP3, GSDMD-N, ASC, C-caspase 1, IL-1β, and IL-18 in HAECs decreased, which inhibited pyroptosis and protected HAECs. Subsequent studies revealed that after low expression of p65, the expression levels of NLRP3 and IL-1β mRNA and pyroptosis-related proteins in HAECs decreased, suggesting that p65 may be the upstream transcription factor of NLRP3 and may promote the expression of NLRP3. This finding is consistent with the previously reported conclusion that p65 binds to the NLRP3 promoter region in nicotine-induced mouse macrophages, and p65 is the upstream molecule of NLRP3 and increases the expression of NLRP3 at the transcriptional level [25]. Immunofluorescence experiments revealed that H_2_O_2_ promoted p65 nuclear translocation and increased p65 transcriptional activity. Previous studies confirmed that the high expression level of NLRP3 in cells is a key factor that activates NLRP3 and stimulates inflammasome activation. Therefore, owing to the regulatory effect of p65 on NLRP3, it can fundamentally regulate pyroptosis and play a vital role. Such notion aligns with the conclusion that the signal provided by NF-κB is necessary for the activation of NLRP3 [23].

When the mechanism of increased p65 expression in HAECs induced by H_2_O_2_ was explored and the target of the protective mechanism against pyroptosis was examined, we found that SIRT1 inhibited the transcription of NF-κB by directly deacetylating the p65 protein at lysine 310 [10]. The expression of SIRT1 deacetylase significantly decreased in H_2_O_2_-mediated HAECs pyroptosis. After high expression of SIRT1, the survival rate of cells increased as detected by PI staining and LDH activity, while the expression of pyroptosis-related proteins was downregulated, which inhibited the inflammatory response and reduced the damage caused by H_2_O_2_ to cells. Therefore, SIRT1 plays a protective role in HAEC pyroptosis. By determining whether SIRT1 directly regulates the acetylation level of p65 and affects the occurrence of pyroptosis of HAECs, we found that H_2_O_2_ promoted the acetylation level of p65 in HAECs. The overexpression of SIRT1 decreased the expression level of p65, and the expression level of acetylated p65 (Lys310) decreased more significantly, indicating that SIRT1 inhibited the level of acetylation of p65. Co-immunoprecipitation experiments revealed a direct interaction between SIRT1 and p65. To verify how SIRT1 regulates the transcriptional activity of p65, immunofluorescence staining was performed, which revealed a significant decrease in the expression of p65 in the nucleus after the overexpression of SIRT1. SIRT1 also inhibited the nuclear translocation of p65, indicating that SIRT1 can partly inhibit the nuclear translocation of p65 by deacetylating p65, thereby inhibiting the transcriptional activity of p65, reducing the expression of NLRP3 and IL-1β, and finally inhibiting the occurrence of HAEC pyroptosis. Previous studies confirmed that the specific acetylation of the p65 site can regulate the biological activity of the NF-κB complex [26]. Acetylation at the lysine 310 site can increase the transcriptional activity of p65, which is necessary for the full transcriptional activity of p65. These findings are consistent with our conclusion that SIRT1 regulates the transcriptional activity of p65 through the deacetylation of p65 (Lys310). In summary, H_2_O_2_-induced HAEC pyroptosis is partly mediated by SIRT1 deacetylation of p65.

To identify an effective target of anti-atherosclerotic therapy through SIRT1, we proceeded to identify the upstream regulatory mechanism of SIRT1. We predicted that a direct target may exist between miR-200a-3p and SIRT1 by Targetscan. Based on increasing evidence, miRNA-related mechanisms are helpful in the treatment of atherosclerosis related diseases [16, 17]. MiR-200a-3p, an important regulator of lipid metabolism, has been widely studied. Yu et al. demonstrated that in lipopolysaccharide-induced human brain microvascular endothelial cells, the expression of miR-200a-3p was upregulated, partly through the induction of ROS to promote the expression of NLRP3 and inhibit the Keap1/Nrf2/HO-1 pathway, ultimately playing a role in the promotion of inflammatory response [27]. In recent years, miR-200a-3p has been found to be involved in many diseases, such as coronary atherosclerosis, myocardial ischemia-reperfusion, myocardial hypertrophy, angiogenesis, and hypoxic pulmonary hypertension [28]. However, whether miR-200a-3p regulates the pyroptosis of HAECs by targeting SIRT1 is unclear.

**Figure 8:**
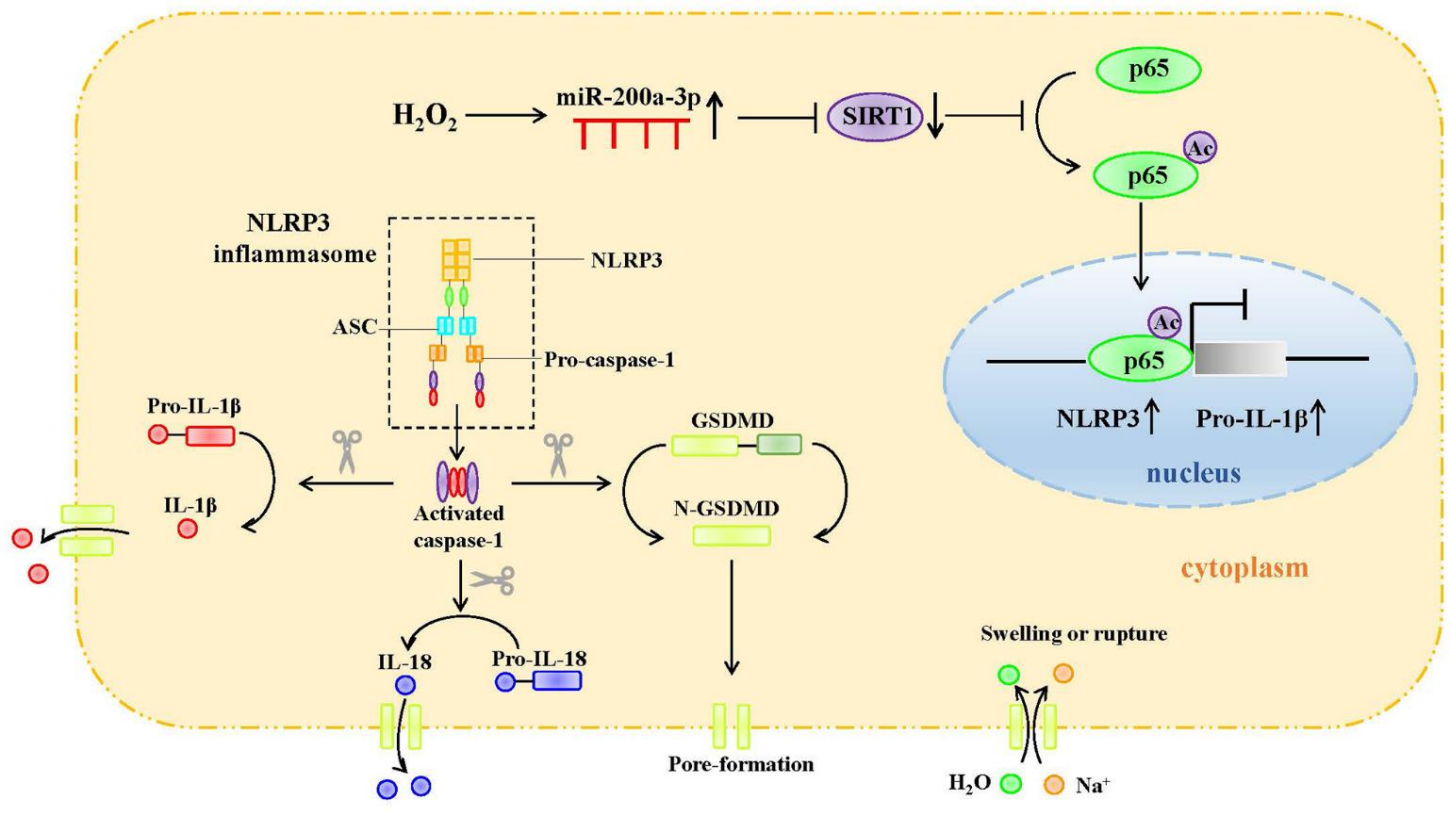
Graphic abstract.

In this study, H_2_O_2_ was found to promote the expression of miR-200a-3p in HAECs. To explore whether miR-200a-3p plays a regulatory role in pyroptosis, after the low expression of miR-200a-3p, the LDH release and positive rate of PI staining cells decreased. Further, the expression level of pyroptosis-related proteins decreased significantly, indicating that the inhibition of miR-200a-3p can prevent the occurrence of HAEC pyroptosis and protect cells. By determining whether miR-200a-3p regulates the expression level of SIRT1, the expression of SIRT1 in HAECs was found to increase significantly after low expression of miR-200a-3p. In contrast, the expression level of SIRT1 in HAECs decreased after the overexpression of miR-200a-3p. These results suggest that miR-200a-3p regulates the expression of SIRT1. Furthermore, binding targets were found between miR-200a-3p and SIRT1 based on bioanalysis. The double luciferase reporter gene experiment verified that miR-200a-3p inhibited the expression of SIRT1. miR-200a-3p sponges the mRNA of SIRT1 and inhibits the expression of SIRT1. By verifying whether miR-200a-3p regulated the pyroptosis pathway by targeting SIRT1, both miR-200a-3p and SIRT1 were expressed at low levels in HAECs. Low expression of SIRT1 was found to significantly reverse the protective effect of the low expression of miR-200a-3p on HAEC pyroptosis and promote the expression of pyroptosis-related proteins. miR-200a-3p has been demonstrated to affect HAEC pyroptosis through the targeted regulation of SIRT1. In this study, we confirmed that miR-200a-3p regulates the expression of SIRT1 and the occurrence of H_2_O_2_-induced HAEC pyroptosis and inflammatory damage, and inhibits miR-200a-3p, which partly plays a role in anti-pyroptosis and reduces cell damage in H_2_O_2_-induced HAECs through the SIRT1/p65/NLRP3 pathway.

Our *in vitro* study revealed the potential role of SIRT1 in H_2_O_2_-induced endothelial cell pyroptosis. H_2_O_2_ downregulates SIRT1, inhibits the deacetylation of p65, and promotes p65 nuclear translocation. P65 mediates NLRP3 transcription and promotes the pyroptosis of HAECs. MiR-200a-3p expression was significantly increased in H_2_O_2_-induced HAECs. The targeted regulation of SIRT1 to inhibit miR-200a-3p reduces the occurrence of cell pyroptosis and protects cells from oxidative stress. Our study provides a new strategy for the clinical prevention and treatment of atherosclerosis.

## MATERIALS AND METHODS

### Mice

Eight-week-old male *apoE^-/-^* mice (C57BL/6 background) were purchased from Changchun Yisi Company. The mice were randomly divided into two groups (n=6/group), the normal chow diet (NCD) group, and the high fat diet (HFD) group. The mice were fed a high fat diet (D12109C, Research Diet: 40 % fat, 1.25% cholesterol, and 0.5% cholic acid) for 12 weeks to establish an atherosclerosis model.

### Cell culture and treatment

HAECs were obtained from Cell Research (Shanghai, China). The cells were grown in Endothelial Cell medium (ScienCell Research Laboratories, USA) supplemented with 5% fetal bovine serum, 1% (v/v) penicillin/streptomycin, and 1% endothelial cell growth factors at 37° C with 5% CO_2_. The cells were digested and passaged with pancreatin (1:250, Solarbio, China). When the cells reached 80% confluence, they were treated with 0.4 mM H_2_O_2_ for 1 h, 2 h, 4 h, or 8 h. To inhibit caspase 1 activity, the cells were treated with the 30 μM of the caspase 1 inhibitor, VX-765 (Beyotime, China), for 1 h.

### Cell viability assay

Cell viability was measured using the Cell Counting Kit-8 (TransGen Biotech, China), according to the manufacturer’s instructions. HAECs were seeded in 96-well plates at a density of 5,000 cells/well and exposed to various concentrations of the compounds for the indicated times. Thereafter, 10 μl of working reagent was added to each well and incubated for 2 h at 37° C. The absorbance was measured using a microplate reader (Synergy HT, Bio-Tek, USA) at a wavelength of 450 nm. Optical density is proportional to the number of living cells in the plate.

### Lactate dehydrogenase (LDH) activity

The LDH activity was measured using an LDH assay kit. For every sample, the working buffer consisted of INX, enzyme solution, and lactic acid solution. A total of 120 μl cell culture supernatant and 60 μl of working buffer were mixed together and incubated at 37° C for 30 min. Finally, the absorbance was measured at 490 nm using a microplate reader.

### Detection of ROS

An ROS assay kit (Beyotime) was used to detect the accumulation of ROS in HAECs, according to the manufacturer’s instructions. Briefly, HAECs were cultured on coverslips in 24-well plates at a density of 2 × 10^4^ cells/well. Cells were loaded with DCFH-DA (10 μM) in serum-free medium in the dark at 37° C for 20 min after pretreatment, and then washed three times with PBS. The fluorescence was examined using a confocal microscope (Olympus, Japan).

### Hoechst 33342/propidium iodide (PI) fluorescent staining

Pyroptosis was assessed using Hoechst 33342/PI double fluorescent staining. HAECs were cultured in 6-well plates at a density of 3 × 10^5^ cells per well and transfected with various constructs or treated with H_2_O_2_. The cells were then stained with 20 μL Hoechst 33342 solution at 37° C in the dark for 10 min, followed by 5 μL PI at 25° C in the dark for 15 min. The stained cells were observed under a confocal laser-scanning microscope (Olympus).

### Cell transfection

HAECs were seeded in six-well plates. When the cells reached 50% confluence, they were transfected with Sirt1 small interfering RNA (siRNA), p65 siRNA, NLRP3 siRNA, ASC siRNA, SIRT1-overexpressing pcDNA3.1-plasmid (100 nM), miRNA-200a-3p mimics, miRNA-200a-3p inhibitor, and negative control siRNA using Lipofectamine 3000 transfection reagent, according to the manufacturer’s instructions. At 48 h after transfection, the cells were treated with H_2_O_2_ for 4 h. Finally, protein expression was determined via western blot analysis, and the gene transfection efficiency was monitored using PCR or western blotting. The sequences of the NLRP3 siRNA used in our experiments are sense, 5′- GUGCAUUGAAGACAGGAAUTT-3′, and antisense, 5′-AUUCCUGUCUUCAA-UGCACTT-3′. The sequences of the ASC siRNA are sense, 5′-GGCAAUCCCACCA-AAUCAUTT-3′, and antisense, 5′-AUGAUUUGGUGG-GAUUGCCTT-3′. The sequences of the RelA/p65 siRNA are sense, 5′- GCACCAUCAACUAUGAUGATT-3′, and antisense, 5′-UCAUCAUAGUUGA-UGGUGCTT-3′. The sequences of the SIRT1 siRNA are sense, 5′-GCUGAUGAACC-GCUUGCUATT-3′, and antisense, 5′- UAGCAAGCGGUUCAUCAGCTT-3′. The sequences of the MiRNA-200a-3p mimics 5′-UAACACUGUCUGGUAACGAUGU AUCGUUACCAGACAGUGUUAUU-3′. The sequences of the miR-200a-3p inhibitor: 5′-ACAUCGUUACCAGAC-AGUGUUA-3′.

### Quantitative real-time PCR (qRT-PCR)

TRIzol reagent (Ambion, USA) was used to extract total RNA from control, treated, and transfected cells. The concentration (ng/μL) of miRNA was quantified and evaluated for purity using a NanoDrop ultraviolet spectrophotometer (Thermo Fisher Scientific, USA). RNA was reverse-transcribed into cDNA using an mRNA reverse transcription kit and an miRNA reverse transcription kit (TransGen Biotech), according to the manufacturer’s instructions. SYBR Premix Ex Taq was used for DNA amplification and an ABI 7500 Fast Real-Time PCR system (Applied Biosystems, USA) was used to quantify the relative RNA expression. After amplification, the threshold cycle (Ct) was determined and the relative mRNA or miRNA levels were calculated using the 2-ΔΔ Ct method. GAPDH was used as an internal control for data normalization. U6 was used to standardize miRNAs. qRT-PCR was performed using the primers listed as follows:

U6 (human) F: 5′-CTCGCTTCGGCAGCACA-3′, R: 5′-AACGCTTCACGAA-TTTGCGT-3′; miR-200a-3p (human): 5′-TAACACTGTCTGGTAACGATGT-3′. SIRT1 (human) F:5′-TAGCCTTGTCAGATAAGGAAGGA -3’, R: 5’–ACAGCTT-CACAGTCAACTTTGT -3′; p65(human) F: 5′-ATGTGGAGATCATTGAGCAGC -3′, R: 5′-CCTGGTCCTGTGTAGCCATT -3′; NLRP3 (human) F: 5’ –GATCTTCGCTG-CGATCAACAG -3’, R: 5’ -CGTGCATTATCTGAACCCCAC -3’; IL-1β(human) F: 5’ -ATGATGGCTTATTACAGTGGCAA -3’, R: 5’ -GTCGGAGATTCGTAGCTGGA -3’; GAPDH (human) F: 5’ -GGAGCGAGATCCCTCCAAAAT -3’, R: 5’ –GGCTGTTG-TCATACTTCTCATGG -3’.

### Western blot analysis

Total protein was extracted from endothelial cells using procedures described elsewhere. High-efficiency RIPA protein lysate (TransGen Biotech) was used to lyse HAECs. The cell lysates were then separated on a 10% or 12% SDS-PAGE gel and transferred onto a PVDF membrane (Millipore, USA). The protein concentrations were determined using the BCA Protein Assay kit (Thermo Fisher Scientific). Thereafter, the membrane was probed overnight at 4° C with primary antibodies against NLRP3 (Abcam), ASC (Abcam), Caspase 1 (Abcam), GSDMD-N (Abcam), NF-κB p65(CST), acetyl-NF-κB p65(Lys310) (CST), SIRT1 (CST), IL-18 (Abcam), and IL-1β (Abcam), which were all diluted at 1:1000 in phosphate buffered saline. Horseradish peroxidase (HRP)-conjugated secondary antibodies (1:2000, Goat Anti-Mouse IgG HRP Affinity Purified PAb, USA; Goat Anti-Rabbit IgG HRP Affinity Purified PAb, USA), goat anti-mouse IgG HRP affinity purified systems, and goat anti-rabbit IgG HRP were applied to the membranes for 50 min at 25° C. Thereafter, the ECL fluorescent developer (Thermo Fisher Scientific) and LAS3000 Imager (Fuji Photo Film Co, Ltd.) were used to detect the protein bands. ImageJ software was used to calculate the intensity of the protein bands. The PVDF membranes were washed with membrane stripping buffer 1 (GenStar, China) for 30 min and then incubated with antibodies overnight. GAPDH (Bioss Technology, China) was used as the internal reference.

### Nuclear and cytoplasmic protein extraction

Nuclear and cytoplasmic protein was extracted from endothelial cells using extraction kit (TransGen Biotech). The endothelial cells were collected and lysed with CPEB I and CPEB II, and supernatant was collected as cytoplasmic protein after centrifugation. CPEB I was added to the precipitate, and the supernatant was discarded after centrifugation. NPEB was added to the precipitate, and the supernatant was collected as nuclear protein after centrifugation. The samples were stored at -20° C for subsequent experiments.

### Coimmunoprecipitation (CO-IP)

Cells were lysed in ice-cold RIPA buffer supplemented with protease and phosphatase inhibitor complexes. Whole cell lysates (400 μl) were incubated overnight with an anti-p65 antibody (6 μl) and IgG (2 μl) at 4° C. Subsequently, 40 μl of protein A/G magnetic bead slurry was transferred to a 1.5-ml tube. The beads were washed three times with PBST, added to whole-cell lysates, and mixed for 2 h at room temperature to yield immune complexes. The bound proteins were eluted via boiling with loading buffer and evaluated via western blotting.

### Immunofluorescence

Immunofluorescence staining was performed to detect the expression of p65 in endothelial cells. Briefly, the cells were fixed with 4% paraformaldehyde for 30 min, penetrated with 0.3% Triton X-100 for 1 h, and then blocked with goat serum for 1 h. Subsequently, the cells were incubated overnight with the anti-p65 antibody at 4° C, followed by Alexa Fluor-conjugated secondary antibody (Invitrogen, USA) in the dark for 40 min. The nuclei were stained with 4′,6-diamidino-2-phenylindole (DAPI, Beyotime) for 5 min. Finally, cells were imaged using a laser scanning confocal microscope (Olympus).

### Luciferase reporter assays

Luciferase reporters containing wild-type or mutated SIRT1 plasmids were constructed using GP-miRGLO vectors (GenePharma, China). The luciferase vector (100 ng) containing the pcDNA3.1-SIRT1 plasmid was co-transfected with miR-200a-3p mimic and negative control (scramble sequence of miR-200a-3p) into human embryonic kidney 293T (HEK 293T) cells using Lipofectamine 3000 (Invitrogen), with 10 ng of Renilla luciferase reporter used as an internal control. After 48 h, the cells were collected and lysed. Luciferase activity was determined using the Dual-Luciferase Reporter Assay System (TransGen Biotech), according to the manufacturer’s instructions.

### Statistical analysis

All experiments were independently repeated at least three times, and the results are expressed as mean ± standard deviation (SD). Statistical analysis was carried out using GraphPad software (version 8.0). The average fluorescence intensity was analyzed using ImageJ. *P*-values were determined using analysis of variance. Statistical significance was set at P < 0.05.
